# Evaluating the Problem of Fraudulent Participants in Health Care Research: Multimethod Pilot Study

**DOI:** 10.2196/51530

**Published:** 2024-06-04

**Authors:** Vithusa Kumarasamy, Nicole Goodfellow, Era Mae Ferron, Amy L Wright

**Affiliations:** 1 Lawrence S Bloomberg Faculty of Nursing University of Toronto Toronto, ON Canada

**Keywords:** fraudulent participants, threats to data integrity, online recruitment, multimethod study, health care research, bots, social media

## Abstract

**Background:**

The shift toward online recruitment methods, accelerated by the COVID-19 pandemic, has brought to the forefront the growing concern of encountering fraudulent participants in health care research. The increasing prevalence of this issue poses a serious threat to the reliability and integrity of research data and subsequent findings.

**Objective:**

This study aims to explore the experiences of health care researchers (HCRs) who have encountered fraudulent participants while using online recruitment methods and platforms. The primary objective was to gain insights into how researchers detect and mitigate fraudulent behavior in their work and provide prevention recommendations.

**Methods:**

A multimethod sequential design was used for this pilot study, comprising a quantitative arm involving a web-based survey followed by a qualitative arm featuring semistructured interviews. The qualitative description approach framed the qualitative arm of the study. Sample sizes for the quantitative and qualitative arms were based on pragmatic considerations that in part stemmed from encountering fraudulent participants in a concurrent study. Content analysis was used to analyze open-ended survey questions and interview data.

**Results:**

A total of 37 HCRs participated, with 35% (13/37) of them engaging in qualitative interviews. Online platforms such as Facebook, email, Twitter (subsequently rebranded X), and newsletters were the most used methods for recruitment. A total of 84% (31/37) of participants indicated that fraudulent participation occurred in studies that mentioned incentives in their recruitment communications, with 71% (26/37) of HCRs offering physical or electronic gift cards as incentives. Researchers identified several indicators of suspicious behavior, including email surges, discrepancies in contact or personal information, geographical inconsistencies, and suspicious responses to survey questions. HCRs emphasized the need for a comprehensive screening protocol that extends beyond eligibility checks and is seamlessly integrated into the study protocol, grant applications, and research ethics board submissions.

**Conclusions:**

This study sheds light on the intricate and pervasive problem of fraudulent participation in health care research using online recruitment methods. The findings underscore the importance of vigilance and proactivity among HCRs in identifying, preventing, and addressing fraudulent behavior. To effectively tackle this challenge, researchers are encouraged to develop a comprehensive prevention strategy and establish a community of practice, facilitating real-time access to solutions and support and the promotion of ethical research practices. This collaborative approach will enable researchers to effectively address the issue of fraudulent participation, ensuring the conduct of high-quality and ethically sound research in the digital age.

## Introduction

### Background

Recruiting participants for health care research through online methods and platforms is becoming increasingly prevalent, particularly in recent years [1-3]. In response to the COVID-19 pandemic in 2020, many researchers had to shift their recruitment strategies from traditional approaches to online methods such as email; listserves; websites; social media platforms such as Facebook, Instagram, and X (formerly known as Twitter); and crowdsourcing websites including Amazon Mechanical Turk and Prolific [1,4-11]. While this sudden shift was unexpected, researchers are recognizing the numerous benefits associated with online recruitment strategies. Web-based recruitment methods offer the potential to reach a broader and more diverse participant pool, including made-marginalized populations [1,3,8,12-19]. They also enable targeted sampling [4,20], offer convenience for participants [3,4,8,14,15,18,19,21], enhance participant anonymity [1,12,14,22], facilitate faster recruitment [8,18,23], and lower recruitment costs [2-4,8,14,15,18,19,21]. Despite its numerous benefits, online recruitment brings with it several challenges. One challenge is the increased risk of fraudulent participants, defined as ineligible persons or computer bots designed to pose as real people to participate in research studies, threatening data quality [3,11,17]. For instance, Pozzar et al [2] investigated the extent of fraudulent participants in health care research using online recruitment methods, specifically social media, and found that almost their entire sample of 271 survey respondents were either fraudulent (94.5%) or suspicious (5.5%). In a qualitative study with 19 research experts, Teitcher et al [24] found that participant misrepresentation was a common problem in online research, with various forms of misrepresentation present, such as duplicate responses, fraudulent demographic information, and dishonesty about eligibility criteria.

Fraudulent participants generally fall into 2 main categories: real humans who participate in a disingenuous manner and computer bots designed to impersonate human participants [18]. Both human and computer bots attempt to participate in research studies for which they are not qualified or attempt to participate multiple times in the same study [17,25]. The presence of fraudulent participants in research studies can lead to various detrimental outcomes. These include the increased financial burden of identifying and addressing the impacts of fraudulent participants as well as the obligation to compensate such individuals, including bots that generate multiple invalid responses, in accordance with the study protocol [26]. In addition, the presence of fraudulent participants can compromise the validity and reliability of research findings, potentially resulting in misguided recommendations that may have harmful consequences [1,3,17].

The presence of fraudulent participants imposes significant stress on research teams [16], such as requiring additional resources to manage and mitigate their impacts. This unforeseen allocation of resources could limit the availability of research staff for other tasks, potentially causing delays in study recruitment and data analysis [16]. Furthermore, fraudulent participants in research studies can lead to the misallocation of funding and human resources through the recruitment and compensation of ineligible individuals [2,16].

Numerous ethical dilemmas that have emerged from this issue include the misuse of research funds to compensate fraudulent participants [1,11,24,27] and implementing invasive participant verification strategies to validate participants’ identities [24]. Underlying ethical principles such as “respect for persons” also come into question as research teams attempt to manage fraudulent participant encounters. For instance, while researchers are required to outline all study methods to individuals interested in participating, researchers are faced with the ethical dilemma of whether they should disclose their fraudulent participant detection and prevention strategies (eg, tracking IP addresses) to potential participants [24]. Although respecting individuals’ rights to be informed about all study components is necessary, researchers also want to avoid deterring anyone from participating in their studies and having fraudulent participants bypass their security measures [24]. The need to adequately balance respect for persons and respect for privacy with data integrity and researcher transparency calls for further attention to ensure that ethical principles are upheld throughout the research process [3,13,24].

Although the issue of fraudulent participants in health care research is not new as health care researchers (HCRs) are increasingly using online strategies to recruit participants, they are becoming more aware of the inherent issues in this approach. Further research to investigate and develop strategies to combat these issues is necessary. One such strategy, the Reflect, Expect, Analyze, and Label (REAL) framework by Lawlor et al [11], provides a structured approach for researchers to assist in the prevention and identification of fraudulent participants within their samples. Researchers are first asked to *reflect* on the inherent vulnerabilities of the recruitment plan and consider built-in survey design elements to avoid fraud. Next, researchers must give thought to the usual patterns in the data that they would *expect* to see and what would present as unusual. The *analyze* stage guides researchers to assess whether actual data patterns align with their expectations. Finally, the framework reinforces the importance of transparency through its *label* stage, encouraging researchers to establish criteria for labeling and excluding fraudulent responses, addressing the issue of underreporting fraudulent participants. While it does not provide an exact count of fraudulent participation, the framework assists researchers in detecting and addressing the inclusion of fraudulent participants, contributing to the mitigation of this challenge. Considering the criticality of upholding research integrity, further investigation is required to identify and address fraudulent participants during the recruitment stages of health care studies in which online methods are used.

### Aims

The aim of this study was to describe the issue of fraudulent participants in health care studies in which online methods and platforms are used to recruit participants to provide HCRs with prevention strategies. We aimed to answer the following research questions: (1) What are the experiences of HCRs when encountering fraudulent participants through online recruitment strategies? (2) How do HCRs identify fraudulent participants? (3) How do HCRs deter fraudulent participants? (4) How do HCRs ensure the integrity of the data they collect when fraudulent participants are suspected?

## Methods

### Design

Using a multimethod sequential approach, we conducted a pilot study to explore the experiences of HCRs with encountering fraudulent participants when using online recruitment strategies. The study consisted of sequential quantitative and qualitative phases involving a web-based survey that informed semistructured interviews that used a qualitative descriptive design.

### Participants and Recruitment

We recruited HCRs from health care–related Faculties, schools, or departments at colleges or universities in Canada who had suspected at least one fraudulent participant in a study conducted in the previous 5 years using web-based recruitment methods. For this study, an HCR was defined as a member of a research team, including principal investigators, coinvestigators, research managers, research coordinators, research officers, and research assistants or some iteration of these roles or job titles. We used purposive and snowball sampling to recruit participants by distributing a digital recruitment flyer via targeted email outreach to individual researchers and mass email distribution by faculty, department, and organization administrators to their researcher employees on our behalf. Potential participants were directed to complete a web-based survey. Our sample size was based on pragmatic considerations, including budget constraints, finding timely solutions for fraudulent participants in our concurrent study, and strategies for pilot studies supported by the literature [28]. As such, we aimed to recruit 30 participants for the quantitative phase of the study and 10 participants for the qualitative arm. Participants who indicated their interest in taking part in a follow-up interview and provided detailed and varied responses to open-ended questions were contacted for an interview.

To safeguard against attracting fraudulent participants, several precautionary measures were implemented. First, a captcha was included at the beginning of the survey to deter automated submissions. In addition, participants were contacted directly through their professional email addresses obtained from their respective employers’ websites. Recruitment was conducted through direct requests made to college or university Faculties, departments, or organization administrators, who in turn sent bulk emails to their staff members to invite participation. To enhance the authenticity of participants, those who consented to a web-based interview were interviewed with their cameras turned on, ensuring a form of verification.

### Data Collection

In the quantitative arm of the study, we used a web-based survey in the REDCap (Research Electronic Data Capture; Vanderbilt University) [29] software platform to collect data using closed- and open-ended questions to identify the circumstances of HCRs’ encounters with fraudulent participants, such as the number of studies in which a fraudulent participant was discovered, and the online platforms and strategies used to recruit participants. REDCap [29] is a secure web application that supports web-based data collection for research studies [30,31]. In the subsequent qualitative arm, participants were interviewed virtually on Zoom (Zoom Video Communications) using a semistructured interview guide to better understand HCRs’ experiences with encountering fraudulent participants and the approaches used to prevent fraudulent participants in future studies. The interview guide was informed by the results of the survey, and the questions were designed to gain a further understanding of the answers provided in the survey. All interviews were recorded with the permission of the participants and lasted approximately 30 minutes.

### Analyses

Triangulation was performed during data analysis and interpretation. We analyzed the quantitative survey data by using descriptive statistics, including means, frequencies, and percentages. Content analysis was then conducted on the interview and open-ended survey data. A total of 3 researchers read the transcripts multiple times to familiarize themselves with the data and decided on the analysis of manifest content—the visible, obvious components of the text [32]—following the methods described by Elo and Kyngäs [33] and Vaismoradi et al [34]. Open coding was then performed, and 3 researchers met several times to categorize and compare codes both with each other and with the entire data set [33,34], and a categorical summary was devised [32-34].

### Ethical Considerations

All procedures were reviewed and approved by the Health Sciences Research Ethics Board of the University of Toronto (44014). Participants provided informed consent before participating in the study, which included the opportunity to opt out of participating. All data was deidentified, using participant codes in place of names or other identifying information. Data were anonymized during the transcription process, with all names, including organizational names, and other possibly identifying information removed from the final transcription. All participants who participated in the study were compensated with a CAD $5 (US $3.67) gift card for completing the survey and an additional CAD $15 (US $11) gift card for participating in an interview.

## Results

### Overview

The study results are presented in an integrated manner, in which findings from both quantitative and qualitative components are combined to facilitate comparison and expansion. Similar results are grouped together, enhancing the comprehensive analysis of the data. A total of 43 individuals accessed and initiated the survey, of whom 6 (14%) were excluded (n=2, 33% did not complete the consent form; n=2, 33% did not complete any part of the survey; and n=2, 33% did not meet the eligibility criteria). This resulted in a final sample of 37 participants for the quantitative arm of the study. Of these 37 participants, 13 (35%) took part in an interview with authors VK or NG. The entire sample had a mean age of 35.8 (SD 9.9) years and held various academic rankings and research positions, including postdoctoral fellows and graduate students, with an average role duration of 4.2 (SD 3.8) years, and most participants (n=13, 35%) were employed at a nursing faculty (refer to Table 1 for a summary of the sample characteristics). It is important to note that Twitter underwent rebranding and became known as X after conducting interviews for this study [7]. Participants refer to Twitter rather than X in the quotations provided.

**Table 1:** Characteristics of health care researchers.

Characteristic			Survey participants (n=37)		Interview participants (n=13)
Age (y), mean (SD)			35.8 (9.9)		39.4 (10.4)
**Age range (y), n (%)**					
	20-29	12 (32)		3 (23)	
	30-39	13 (35)		4 (31)	
	40-49	6 (16)		3 (23)	
	50-59	6 (16)		3 (23)	
**Research role, n (%)**					
	Pretenure professor^a^	10 (27)		6 (46)	
	Tenured professor^b^	4 (11)		2 (15)	
	Research assistant^c^	10 (27)		3 (23)	
	Research manager^d^	7 (19)		2 (15)	
	Lead researcher^e^	3 (8)		0 (0)	
	Graduate student^f^	2 (5)		0 (0)	
	Postdoctoral fellow	1 (3)		0 (0)	
Years in the role, mean (SD; range)			4.2 (3.8; <1-17)		5.0 (4.7; <1-10)
**Research department, n (%)**					
	Nursing	13 (35)		6 (46)	
	Medicine	7 (19)		2 (15)	
	Health sciences	5 (14)		0 (0)	
	Public health	4 (11)		2 (15)	
	Social work	3 (8)		2 (15)	
	Pharmacy	2 (5)		1 (8)	
	Psychology	1 (3)		0 (0)	
	Research institute	1 (3)		0 (0)	
	Social science	1 (3)		0 (0)	

^a^The pretenure professor role includes assistant professors.

^b^The tenured professor role includes professors and associate professors.

^c^The research assistant role includes the research coordinator. If a participant indicated that their role was both research assistant and PhD student, they were counted as a research assistant.

^d^The research manager role includes project officer, program manager, laboratory manager, assistant director, research officer, and research administrator.

^e^The lead researcher role includes scientist, coinvestigator, and staff scientist.

^f^The graduate student role includes PhD students.

### Approaches to Recruitment and Data Collection in Studies With Suspected Fraudulent Participants

Findings revealed that 46% (17/37) of HCRs reported suspicions of fraudulent participants in qualitative studies only; 24% (9/37) did so in quantitative studies only; and a further 30% (11/37) did so in mixed methods or qualitative and quantitative studies (Table 2). Most HCRs (24/37, 65%) suspected fraudulent participants in a single study. Facebook, email, Twitter, and online newsletters were the most used online methods or platforms. In recruitment communications, incentives were mentioned in 84% (31/37) of cases, and data collection methods were provided in 70% (26/37) of communications. Online surveys were the most widely used data collection method, and email was the predominant method for expressing interest in participation.

**Table 2:** Research approaches in studies with fraudulent participants.

		Survey participants (n=37), n (%)	Interview participants (n=13), n (%)
**Number of studies with suspected fraudulent participants**			
	1	24 (65)	7 (54)
	2	6 (16)	2 (15)
	3	6 (16)	3 (23)
	4	0 (0)	0 (0)
	5	1 (3)	1 (8)
**Online method or platform used to recruit participants**			
	Facebook	26 (70)	9 (69)
	Email	24 (65)	8 (62)
	Twitter (subsequently rebranded X)	24 (65)	7 (54)
	Online newsletter	21 (57)	8 (62)
	Instagram	15 (41)	4 (31)
	LinkedIn	9 (24)	3 (23)
	Paid advertisements (eg, Google or Facebook advertisements)	9 (24)	3 (23)
	Other	15 (41)	6 (46)
**Information included in recruitment communications**			
	Mention of incentive	31 (84)	12 (92)
	Data collection method	26 (70)	11 (85)
	All inclusion criteria	24 (65)	10 (77)
	Some inclusion criteria	14 (38)	2 (15)
	Link to an online survey	14 (38)	5 (38)
	No incentive provided	6 (16)	2 (15)
	Other	2 (5)	0 (0)
	None of the inclusion criteria	1 (3)	0 (0)
**Data collection methods**			
	Online survey	24 (65)	9 (69)
	Online interview	22 (59)	9 (69)
	Phone interview	11 (30)	5 (38)
	Online focus group	8 (22)	3 (23)
	In-person interview	3 (8)	1 (8)
	Paper-and-pencil survey	3 (8)	2 (15)
	In-person focus group	1 (3)	1 (8)
**Methods used by participants to express interest in taking part**			
	Email	30 (81)	12 (92)
	Link to web-based survey	19 (51)	7 (54)
	Phone	13 (35)	6 (46)
	Social media message	6 (16)	4 (31)
	Other	3 (8)	0 (0)
**Research methodology used when fraudulent participants were discovered**			
	Qualitative	17 (46)	5 (38)
	Quantitative	9 (24)	3 (23)
	Both qualitative and quantitative	11 (30)	5 (38)

In terms of incentives provided to study participants, most HCRs (26/37, 71%) offered physical or electronic gift cards, whereas <4% (1/37, 3%) offered the chance to win a prize, such as Apple AirPods. Gift card values ranged from CAD $5 (US $3.67) to CAD $100 (US $73.39), with draw prizes having higher values (eg, AirPods valued at CAD $250 [US $183.48]). Some HCRs used a staggered compensation approach offering gift cards for survey completion and higher-value compensation for participation in interviews or focus groups. One HCR allowed participants to choose between a gift card or an e-transfer for compensation.

### Fraudulent Participation Tactics

HCRs speculated about the tactics used by fraudulent participants to take part in research studies. One HCR speculated that fraudulent participation may be linked to the use of artificial intelligence (AI) technology that scanned the internet for recruitment advertisements, including compensation indicators such as the dollar sign ($). Another HCR recounted an incident she described as “scary,” when the research team received 17 fraudulent emails shortly after their partner organization sent a recruitment email to their own listserve. This participant reasoned that the listserve had somehow been accessed and infiltrated by bots.

Individuals attempted to participate fraudulently by using various tactics, such as falsely claiming to reside within the study catchment area and asserting eligibility criteria related to race, specific health conditions or certain professional designations. For example, one HCR shared their observations about participants who pretended to be both a youth and a parent, representing 2 distinct target populations within the same study.

HCRs suggested that fraudulent participants may have searched for web-based information before their interview to convincingly respond to interview questions, such as familiarizing themselves with the responsibilities of an eligible individual (eg, a prenatal nurse). During some interviews, unusual pauses and typing sounds were observed, suggesting to one HCR that fraudulent participants may have been searching for web-based answers during the interview. An HCR described their experience as follows:

There was another thing to where we noticed that sometimes there would be pauses and typing sounds on the computer. So, it’s almost like the person is Googling things or, you know, uncertain about certain things. It’s like you shouldn’t need to Google the city you’re in.

### Suspicions and Discovery of Fraudulent Participants

HCRs encountered various unusual behaviors and circumstances that raised suspicions of fraudulent participation across different study methodologies. Notably, suspicions were most common during online surveys (25/37, 68%), video interviews (23/37, 62%), phone interviews (12/37, 32%), and online focus groups (9/37, 24%). In email communications, HCRs observed discrepancies between participant names and email addresses, unusual timing of emails (eg, early morning), or receiving a batch of emails at the exact same time or within a short time frame. Some emails lacked the typical contextual information, such as how participants learned about the study or their eligibility, whereas other emails were excessively lengthy or nonsensical and included the use of profanities and explicit sexual content in survey responses.

HCRs also suspected or discovered fraudulent participants when they used different names within a string of email interactions (such as using the name Nancy in the first email and the name Sarah in a subsequent email), stereotypical Western names such as John Smith, or the names of famous people (eg, Britney Spears). Multiple signatures of different names on the same consent form and an unusually high representation of marginalized groups raised doubts about participant authenticity. HCRs noted other peculiar participant behaviors, such as responses that would seem to suggest that the participant had a complete lack of understanding of or familiarity with the study topic or stating that one’s city of residence was “Ontario,” which is a province, not a city, in Canada. Background laughter and statements such as “oh, forget it” and logging off further reinforced the impression that these individuals were not fully committed to the research, behavior that, according to HCRs, was atypical in an individual from the target population.

HCRs grew skeptical of participants’ authenticity when participants would not disclose their mailing address despite researchers’ explanations that a mailing address was required to receive the study compensation. HCRs also raised concerns about the identity of participants in instances in which they refused to appear on camera during interviews. This behavior raised suspicions when participants did not possess the expected vocal characteristics associated with their claimed demographic, such as one participant claiming to be a child but having a voice that sounded like that of an adult. One HCR who encountered fraudulent participants who provided survey responses that did not make sense or were unrelated to the question or completed questions in an unusually quick time was prepared for the possibility of fraudulent participants by using a recruitment website that allowed for the tracking of the amount of time that a respondent takes to complete survey questions or activities.

Several HCRs spoke about detecting fraudulent participants due to surges in emails or completed surveys after the use of Facebook [6] and X [7] in particular and, at times, LinkedIn [35] to publish a recruitment advertisement. These surges caused some HCRs to stop using these social media platforms to recruit. One participant was aware of the possible issues with social media platforms and, therefore, avoided their use altogether:

The worst we saw was not LinkedIn, although LinkedIn also attracts [fraudulent participants], but we noticed spikes every time we would put it on Facebook or Twitter. In fact, the cannabis study, I stopped advertising on Twitter and Facebook because we were getting nothing that was eligible, and we were just bombarded with ineligible responses.

Surges in fraudulent participants were not limited to Facebook and X. Paid-for services such as Honeybee Health [36] (a digital clinical trial recruitment platform), Qualtrics [37] (a web-based survey platform), and paid-for advertisements on Facebook did not prevent the infiltration of fraudulent participants. HCRs also spoke about puzzling circumstances such as emails from several respondents within a short time frame and these respondents attempting to schedule their interview quickly, which, according to one participant, was unlikely considering that their target population, prenatal nurses, are very busy and typically require some time to schedule an interview. One HCR encountered inconsistencies related to participants’ birth dates in a longitudinal study. As part of the study, participants were asked to provide their birth date in multiple surveys over time. To the HCR’s surprise, some participants provided different birth dates across these surveys.

In total, 15% (2/13) of the HCRs shared their notable experiences with participants who raised questions regarding the details of compensation. Specifically, participants inquired about whether the compensation would be in the form of a gift card and whether it would be sent via mail or electronically, which, according to the HCRs, was unusual. Some participants expressed a strong insistence on receiving an electronic gift card from specific companies such as Amazon even when the HCRs were offering gift cards from different companies such as Tim Hortons or Starbucks or when no gift card was being provided as compensation at all. Finally, participants provided responses to open-ended questions that, ultimately, had HCRs questioning the validity of the responses:

And then of course, reading their qualitative responses because some of the questions...were open-ended, and the responses also just did not sound like responses we might have gotten from a real parent.

### Treatment of Fraudulent Participants and Their Data

Once research teams suspected or identified fraudulent participants, they implemented various actions to address the problem. One common action was consulting and discussing participant concerns with other research team members, including decision-making regarding the appropriate handling of data collected from suspected fraudulent participants and additional verification processes. For instance, to assess participant eligibility, some HCRs conducted follow-up phone calls with suspected participants to ask additional screening questions in the hope that the research team could obtain a better sense of participants’ eligibility. Some researchers requested proof of identity, such as photo identification or professional license number, to verify participants’ identity and adherence to the inclusion criteria. One HCR described that they used a screening protocol to classify responses from suspected fraudulent participants:

We coded all responses as “complete” or “bad” (ineligible); we also had a “PARTIAL” status for those who did not complete the questionnaire before the study was closed.

While most HCRs informed suspected fraudulent participants of their ineligibility, some HCRs chose to cease all communication, including not responding to emails, scheduling interviews, or sending survey links. In addition, all HCRs excluded data from fraudulent participants in their data analysis, often storing data in a separate file. A total of 15% (2/13) of the HCRs expressed interest in conducting a secondary analysis or separate study using the collected data from fraudulent participants with the intention of deepening their understanding of this emerging issue and facilitating open discussions among other HCRs regarding their experiences with fraudulent participant encounters.

### Strategies Used to Prevent Fraudulent Enrollment, Identify Fraudulent Participants, and Verify Their Identity

HCRs explored various strategies to prevent the fraudulent participation of individuals in their studies and safeguard the integrity of their findings. While some HCRs implemented specific measures, others reflected on lessons learned and discussed strategies they would use in subsequent studies. Multimedia Appendix 1 provides a detailed list of these strategies.

One key approach to preventing the participation of fraudulent individuals involved incorporating additional security features such as a captcha into survey platforms to deter fraudulent participation. A captcha [38] is a widely used method that helps verify the authenticity of participants by requiring users to complete a task or answer a challenge that is easy for humans to perform but difficult for automated computer programs (bots) to solve. HCRs also engaged in investigation of the authenticity of participants, whether prompted by their suspicion or as a preemptive strategy, by using other survey platform features to track IP addresses, geolocation, latitude and longitude, and participants’ postal codes when they discovered that geographic markers or indicators did not match the participants’ stated location of residence. One research coordinator stated the following:

And then when you match up the location and the postal code, sometimes there’s a mismatch in terms of they say that they’re in Toronto, but then the postal code starts is the V, which is in out of Vancouver.

Other strategies were used to discourage fraudulent participation, including selectively using social media platforms or groups instead of advertising broadly and publicly, avoiding specific symbols or words such as the dollar sign that could be detected by AI systems, and refraining from explicitly mentioning incentives in recruitment advertisements. As stated by an assistant professor from a Canadian university, “...we removed the survey link from study advertisements to prevent any participant from filling it out without eligibility.”

When emails were used to communicate with potential participants, HCRs analyzed emails for specific patterns indicative of fraudulency, such as duplication (multiple survey entries from the same email address or duplication of text within emails even if the addresses were different) as well as their content to ensure that the language matched researchers’ expectations. Once fraudulent participation was suspected, some HCRs introduced additional requirements for participants to provide personal information or verify their identity, such as full names, photo ID, mailing addresses, or professional or institutional email addresses, as an additional deterrent against fraudulent participation.

HCRs also highlighted the need for comprehensive screening protocols that go beyond eligibility screening and are integrated into the study protocol, grants, and research ethics board (REB) applications. They underscored the importance of attentiveness to participants’ verbal and nonverbal cues to assess their sincerity, genuineness, and ultimate authenticity. Concurrent analysis of data was also recommended to identify the potential for fraudulent participation to mitigate further issues with recruitment and data collection and safeguard data integrity.

When fraudulent participants were successful at passing eligibility criteria, some HCRs still requested participants to verify their identity. When participants refused to turn on their camera, this was often seen as a red flag:

[W]e did at the beginning, and we did it with some people that pushed us a little bit ‘cause there were a few that pushed. And as soon as we said that they had to turn on their camera and show photo ID, they never followed through.

### Motivation for Participating Fraudulently

HCRs discussed the potential motives behind individuals choosing to partake in research despite not meeting the specified criteria for inclusion. Several participants emphasized the role of incentives as a significant driving force. One researcher expressed astonishment at the discovery of individuals purposefully participating in a fraudulent manner, fully aware that the receipt of the CAD $100 (US $73.39) gift card was not guaranteed. It was surprising to the researcher that these individuals would willingly invest their time in engaging in online interviews and completing surveys, all for the mere possibility of receiving the gift card. Another participant shared her experience of a participant who expressed intentions to encourage every member of her immediate family to participate in the project even though her mother did not meet the inclusion criteria. The researcher went on to contemplate the benefits and challenges associated with offering financial incentives to participants. On the one hand, providing compensation demonstrates to participants that researchers value their time and effort. However, it may inadvertently encourage individuals to misrepresent themselves to obtain compensation:

I think the incentives are always a problem and a blessing. You do want to compensate people for time, but at the same time, you don’t know people’s motivations for why they do certain things and what their needs are. And especially during these times of inflation. And for somebody, [a] $30 gift card to a grocery store could make a difference in one week depending on the type of nutrition they’re going to consume.

The use of online platforms to recruit participants offers a level of anonymity and convenience that may not be readily achievable in traditional in-person recruitment and research settings. This aspect was highlighted by an HCR who explained how online participation allows individuals to engage in interviews without the need to turn on their camera. In addition, participants can easily self-screen by simply clicking “a button” rather than being screened by a researcher who might uncover their ineligibility to participate.

HCRs speculated about the potential occurrence of fraudulent participation driven by personal amusement. One HCR shared about their research team’s encounters with individuals displaying peculiar behavior during interviews, such as hanging up in the middle of the interview, joking around, making funny remarks, and background laughter. The researcher likened these situations to Halloween or prankster activities, such as “knocking on people’s doors and running away or toilet papering someone’s house.”

### Ethical and Practical Challenges of Fraudulent Participants

Identifying fraudulent participants presented significant resource challenges, especially in studies with high participant interest. An HCR highlighted the extensive resources needed to identify and exclude fraudulent participants from their study:

We used LinkedIn to recruit, and within the first week of posting this ad, making it live, I got about 200 emails from potential participants, and I couldn’t distinguish between imposters, between bot robots, between fraudulent participants and actual participants. So, we had to go back to the drawing board to decide how to deal with this situation because obviously we couldn’t—our sample size was 45, we had 200 people responding to the ad, so we had to narrow it down somehow.

This issue not only caused delays in study timelines but also placed a strain on study budgets. Research teams had to allocate additional resources to effectively manage this challenge, which poses a significant setback for studies with limited funding.

The presence of fraudulent participants posed a considerable source of stress for research teams, with several HCRs expressing limited knowledge of best practices on how to effectively address this issue. This was particularly pronounced in studies involving participant interviews. One participant noted the predominant focus of existing literature on preventive strategies applicable to quantitative research, suggesting a potential hesitation or lack of awareness of qualitative researchers to openly discuss these issues. The lack of transparency surrounding this problem contributed to the limited guidance available for research teams facing similar challenges.

Researchers often faced an ethical dilemma regarding the compensation of individuals identified as fraudulent participants. Some researchers chose not to provide an honorarium to these participants as they provided invalid data, particularly when survey responses indicated a high presence of bots. However, certain researchers decided to give an honorarium to fraudulent participants to preempt potential legal issues. Justifying this decision, one HCR explained that their REB protocol required providing an honorarium to all participants, including those identified as fraudulent. Nevertheless, the issue of compensating ineligible individuals was a subject of debate among HCRs, with frustrations expressed regarding diverting funds from individuals with genuine lived experiences or in need of financial support. One participant highlighted the following:

[I]t’s taking away money from people with lived experience who really need this money and research funding is limited. It’s not like I have unlimited funds.

HCRs raised another ethical concern about the potential invasiveness of verifying eligibility. While requesting proof of identification could help minimize the risk of enrolling fraudulent participants, researchers also struggled to justify requesting proof of identity in their REB protocols as obtaining participants’ personal information did not directly contribute to the study beyond verification purposes. HCRs also viewed a stringent screening process as potentially insensitive, difficult to access, and discouraging for prospective participants. An HCR recruiting individuals with disabilities expressed concerns about the potential harm involved, stating the following:

But it feels a little bit like I’m asking people to confess that they have a disability, and they have to prove it to me that they’re disabled. And that is such a hurtful and potentially harmful thing, right?

Furthermore, HCRs encountered challenges in distinguishing between fraudulent participants and eligible ones, leading to feelings of doubt and guilt. The subjective nature of relying on a researcher’s intuition further complicated the identification process, making it difficult to determine which participants should be included or excluded from a study. The limited interaction between HCRs and participants recruited from online platforms added another layer of complexity in understanding participants’ true intentions.

## Discussion

### Principal Findings

Study findings highlight the pervasiveness of fraudulent participation [12,16] across health care studies involving online recruitment methods regardless of the research methodology, recruitment methods, social media platforms, incentives, and data collection techniques used, underscoring the complexity and ubiquitous nature of this problem. While online methods and platforms, including email; project websites; and social media platforms such as Facebook, Instagram, X, and LinkedIn, offer several benefits such as convenience [3,4,14,16,19], cost savings [2-4,8,14,15,18,19,21], and the opportunity to reach diverse populations [3,8,13-19], they also expose HCRs to risks related to fraudulent participants [2,23,24]. These risks encompass the greater difficulty in determining eligibility [3,4,14] and collection of false data, which can significantly alter results and invalidate findings [3,12,15,17,26], leading to inappropriate and harmful applications [17] as well as the waste of time, funding, and human resources [16,26]. Furthermore, our study highlights the growing challenge of AI and bots in health care research involving online recruitment methods and HCRs’ relatively limited understanding of the various technologies and their capabilities. For instance, circumstances reported by HCR participants that were attributed to AI may, in fact, have been carried out by web-crawling bots (ie, spiderbots) scanning the internet for recruitment advertisements [39-41]. Despite the significant challenges posed by fraudulent participation, the use of online methods and platforms for recruitment offers substantial benefits [1,2,4,8,12-18,20,23] that typically outweigh the associated risks. Therefore, researchers using online strategies to recruit must proactively develop a comprehensive protocol to prevent and detect fraudulent behavior (eg, the REAL framework approach by Lawlor et al [11] to addressing survey fraud) [2,11]. Such a protocol, detailed in the Recommendations section and summarized in Textbox 1, will help ensure the integrity and validity of the research by effectively addressing the challenges posed by fraudulent participants. It is important to note that these recommendations are based on the experiences of a limited number of HCRs.

**Recommendations for preventing and addressing fraudulent participation in health care research involving online recruitment methods.**

**Targeted recruitment**

- Implement targeted recruitment strategies, such as posting study advertisements in closed or topic-specific groups on social media platforms.
- Establish partnerships with relevant organizations that have access to eligible research participants and recruit directly from those groups.
- Disclose only essential eligibility criteria in study advertisements, providing enough information to attract individuals from the target population.
- Do not include the survey link in study advertisements. Instead, require potential participants to contact the research team for further information and screening.
- Avoid using research- or incentive-related keywords in recruitment advertisements.
- Replace symbols in email addresses with their spelled-out equivalents, such as replacing the at sign (@) with the word spelled out to mitigate the risk of automated systems harvesting email addresses for fraudulent purposes (eg, “researchlab at utoronto dot ca”).

**Identification and verification**

- Collect comprehensive participant information, including full name, phone number, mailing address, photo identification, or professional email addresses.
- Use direct communication methods such as phone or video for participant screening rather than email-only screening.
- Observe participant behavior during the screening process, including factors such as response time and nonverbal cues.
- Pay attention to participants’ confidence levels and consistency in their responses.
- Implement a requirement for participants to briefly appear on camera during online interviews for identification purposes.

**Email communication**

- Use email verification services to identify and scrutinize temporary email addresses.
- Monitor and track duplicate email addresses (same email address used by multiple interested participants).
- Examine the format and structure of email addresses, paying attention to generic names followed by a seemingly random combination of letters or numbers.
- Evaluate emails for coherence and correct syntax, ensuring that the email content aligns with the typical language and syntax patterns used by the sample population.

**Incentives**

- Minimize disclosure of financial incentives (type and value) in recruitment materials.
- Avoid the $ sign in recruitment materials.
- Refrain from including research- or incentive-related keywords or hashtags in recruitment advertisements and on social media.

**Survey platform tools and features**

- Use a verification service such as a captcha or TransUnion’s TLOxp [3] to verify human participation and identity and deter automated bots.
- Include honeypot questions (ie, questions that only bots can see), allowing for the identification of potentially fraudulent activity when a bot responds to these questions.
- Track IP addresses, geolocation, or latitude and longitude data to detect suspicious or inconsistent participant locations.

**Question formatting**

- Include checking questions or attention questions (eg, at the end of a question, instruct participants to choose option C in a multiple-choice question).
- Include open-ended questions to assess participants’ knowledge and gauge their familiarity with the research topic.
- Pose specific questions that only legitimate participants would be able to answer accurately.

**Postsurvey checks**

- Analyze when surveys are completed, paying attention to those completed during unusual hours.
- Note the time spent completing surveys to compare to average completion time and identify abnormally fast or slow responses.
- Scrutinize a surge of surveys completed at approximately the same time.

**Analysis of survey responses**

- Monitor incoming data, such as IP addresses and completion locations, to identify unusual patterns.
- Conduct concurrent data collection and analysis when feasible to identify fraudulent participants early and take mitigation measures.
- Select and review survey responses to detect any inconsistencies or suspicious behavior at several points during data collection.

These recommendations provide HCRs with strategies that may be beneficial in preventing or identifying fraudulent participants when using online methods and platforms for recruitment. However, not all recommended measures may be suitable for all studies, and they should be considered within the context of the study goals and how each measure may impact their expected results. Furthermore, HCRs should also be cognizant that, although strategies may deter some fraudulent participants or bots, they are not foolproof as individual scammers and technology are rapidly changing and advancing (eg, closed Facebook groups may contain fraudulent members, participant voices in phone calls or video calls make be “deep fakes” [AI-generated faces and voices] [42], avoiding symbols [eg, $ and @] may only deter simple bots, and tracking IP addresses may be limited due to the increased use of virtual private networks).

Our study sheds light on the various peculiar and irregular behaviors that indicate the presence of fraudulent participants in research. However, HCRs cannot rely on any single indicator of fraudulent behavior to determine the existence of fraudulent participation. Instead, they must be aware of and consider several behaviors and circumstances simultaneously that, together, are a good indication of the presence of fraudulent participants. These behaviors include the use of unusual or very generic Western names, discrepancies between names and email addresses, attempts to negotiate incentive type or delivery, perceived lack of interest in the interview, use of temporary email addresses, surges in emails at unusual times, and inappropriate or outdated terminology, among others. HCRs should also be aware that the absence of incentives or their perceived low value (eg, CAD $5 [US $3.67] Amazon gift card) from recruitment materials may not deter individuals from engaging in inauthentic behavior to obtain compensation [43]. This is particularly true if a fraudulent participant works for or is part of a sophisticated operation that uses AI to systematically search the internet for research studies aiming to accumulate incentives across multiple studies [43]. Our study underscored this phenomenon as one HCR speculated on the involvement of such operations. In addition, in a 2019 blog post, the founder of a market research company highlighted the discovery of a website specifically designed to train individuals in fraudulently completing large volumes of web-based surveys [44]. It is important to note that, while an operation using AI may indeed be more sophisticated, this example may not capture the full spectrum of fraudulent activity. Web crawlers (spiders or spiderbots) can search for information such as studies without advanced AI capabilities [39-41]. Furthermore, individuals may also use manual methods to scour the web in pursuit of accumulating incentives.

HCRs experience ethical conflicts for being too stringent and invasive in participant screening processes, financially compensating fraudulent participants, and potentially excluding legitimate participants from studies. It is recommended that all ethical considerations be outlined and addressed in the study’s REB application and consent form [45] to ensure transparency regarding the steps taken to minimize this issue. In REB applications, researchers need to indicate the amount and type of personal information obtained from participants as part of the screening process, how teams will screen for fraudulent participants, and whether an incentive will be provided to those identified as fraudulent. In addition, research teams should consider stating in their study letter that any participant who is discovered to be providing false or misleading information about their identity may forfeit the incentive despite the time or effort contributed to the study. By considering the diverse tactics used by individuals attempting to participate fraudulently in research and recognizing the significance of incentives regardless of their perceived value, researchers can address the ethical dilemmas uncovered in this study.

### Recommendations

#### Comprehensive Prevention Strategy

As demonstrated by Lawlor et al [11], to effectively prevent and address fraudulent participation in research studies, it is crucial to implement a comprehensive strategy that encompasses prevention, identification, and response measures (Textbox 1). This strategy should involve the use of various security features available on survey platforms to mitigate the risk of fraudulent enrollment. Incorporating tools such as a captcha [3,38] and other security features acts as an initial defense against computer bots attempting to gain access to research studies [2,4,12,26]. It is important to note that, while these security measures are valuable, they may not provide absolute protection against fraudulent participation as technology continues to evolve [1,26].

To minimize the attraction of fraudulent participants, researchers can use a targeted online recruitment approach in addition to security measures [26,46]. This approach involves avoiding the explicit publication of incentives [1,17,24,46], not using some symbols such as the dollar sign, and publishing recruitment advertisements in closed social media groups rather than public ones.

Furthermore, when using online recruitment methods, HCRs should operate under the assumption that some fraudulent participants may successfully bypass security features. To effectively identify such participants, it is recommended to use multiple strategies in the survey design. This includes incorporating honeypot questions, which are hidden questions detectable only by bots [9,26,47], and attention-checking questions that individuals are required to answer as a way to distinguish between high- and low-quality data [8,19,26] and using open-ended questions to assess the genuineness of responses [2,9,27]. Researchers can also leverage additional online survey features such as tracking IP addresses, which can be cross-referenced with other data points such as postal or zip codes to verify participant eligibility and detect duplication [18,24,48]. By monitoring survey entries for time stamps, researchers can identify clustered entries and assess the time taken to complete surveys compared to the average response time [2,23,43].

In addition to survey design considerations, it is crucial to pay attention to key email markers that may indicate fraudulent participation. These markers include duplicate addresses, suspicious email formats (eg, name1234@gmail.com), and temporary emails [12,16,26]. It is worth noting that screening for fraudulent participants requires considerable time and resources. To streamline the identification process, HCRs may opt to classify survey entries into 3 distinct categories: authentic, suspicious, and fraudulent entries [48]. This categorization approach can be particularly useful when dealing with large volumes of surveys, allowing researchers to focus their attention on reviewing entries that raise suspicion.

#### Communities of Practice in Academia

In addition to practical benefits, a community of practice focused on research strategies and ethical considerations when using online recruitment and data collection methods provides a supportive environment where researchers can openly share their experiences and learn from one another. A community of practice is a group of people who share a common concern, a set of problems, or a passion for a particular topic [49]. Through continuous interactions, the group engages in activities that facilitate the deepening of their knowledge and expertise in that area [49]. This collaborative approach not only supports acknowledging the existence and pervasive nature of fraudulent participation but also fosters a culture of collaborative learning, sharing, and continuous improvement. By engaging in ongoing discussions and interactions with colleagues in similar situations, HCRs can refine their prevention strategies, adapt to emerging trends, and contribute to the integrity and validity of health care research in the digital age.

By establishing a community of practice, researchers can bridge the gap between research and practice, allowing for timely access to practical knowledge and insights by sharing experiences, exchanging information, and cocreating effective prevention strategies to stay ahead of the ever-evolving tactics used by fraudulent participants [49-51]. Furthermore, a community of practice serves as a mechanism to collaboratively address the urgent need for conducting research with high-quality data that are free from the influence of fraudulent participation. This proactive approach not only ensures the credibility and reliability of health care research but also fosters a culture of continuous learning and improvement within the academic community.

### Limitations

This study has several limitations. First, the data collected for this research are limited to Canada. While this provides valuable insights into fraudulent participation in the Canadian context, it may not fully capture the variations and complexities of fraudulent behaviors in different countries and cultural settings. Second, it is important to note that these recommendations stem from a pilot study involving a limited number of HCRs. Therefore, while these suggestions can be valuable for researchers conducting similar studies, the generalizability of the recommendations is limited to general population recruitment. Furthermore, we did not explicitly collect data on the HCRs’ level of expertise with online recruitment strategies or data describing the samples that the HCRs were recruiting, such as whether they were recruiting health professionals, patients, or the general population. This is an area that should be explored in future research with a more extensive design to explore these aspects in greater depth to support the potential approaches and adaptations during the recruitment process. Similarly, specific data were not collected on whether participants were describing secondhand accounts reported to them by research staff or whether they were involved firsthand in the day-to-day detection of fraudulent participants. Future research with a larger sample size may delve deeper into these nuances. Third, due to the inherently narrow scope of our pilot study, there is a need for studies of a larger scope in other countries and scientific fields and with larger sample sizes to gain a more comprehensive picture of fraudulent behaviors and the strategies to prevent, deter, and identity fraudulent participants. In addition, collaborating with IT and security specialists would help build effective mitigation strategies. Finally, this study did not include direct input from fraudulent participants themselves. While our study findings provide the reader with insights into potential motivations for fraudulent participation based on the experiences of researchers, not having direct access to the perspectives of these participants may limit the depth of understanding regarding their underlying motivations and tactics. Future research that incorporates the voices of fraudulent participants could provide valuable insights and enhance the development of more targeted prevention and mitigation strategies in studies involving online recruitment methods and platforms.

### Conclusions

In conclusion, this study sheds light on the complex and pervasive problem of fraudulent participation in health care research when online recruitment methods are used. The findings emphasize the need for HCRs to be vigilant and proactive in identifying, preventing, and responding to fraudulent behavior. To address this challenge effectively, HCRs must go beyond relying on intuition and subjective methods that may introduce bias. Instead, they should develop and implement several prevention, verification, and mitigation strategies at once and not rely on post hoc measures to verify participant data. In addition, researchers should stay informed of the ever-changing landscape of the internet and the technology and methods used by fraudulent participants to bypass safeguards. By understanding that the driving force for deception may be the prospect of gaining a financial incentive regardless of its value and recognizing the diverse tactics used by fraudulent participants to gain compensation, researchers can be prepared to encounter and effectively manage this problem by developing and implementing robust prevention and management strategies. HCRs should be encouraged to document the evidence of fraud within their studies, providing sufficient information for other researchers to become aware of the potential dangers when using online research methods and to establish their credibility and protect the integrity of their research. To address the challenge of fraudulent participation in a timely manner, researchers can establish a community of practice to access timely solutions and support, enabling them to address the problem more effectively and conduct high-quality and ethically sound research. By taking collective action with other researchers and staying informed, researchers can safeguard the integrity and validity of health care research in the digital age.

## Appendix

Multimedia Appendix 1 Indicators leading to suspicion of fraudulent participants in health care research.

## Abbreviations

AI: artificial intelligence
HCR: health care researcher
REAL: Reflect, Expect, Analyze, and Label
REB: research ethics board
REDCap: Research Electronic Data Capture

## References

[1] Griffin M, Martino RJ, LoSchiavo C, Comer-Carruthers C, Krause KD, Stults CB, Halkitis PN (2022). Ensuring survey research data integrity in the era of internet bots. Qual Quant.

[2] Pozzar R, Hammer MJ, Underhill-Blazey M, Wright AA, Tulsky JA, Hong F, Gundersen DA, Berry DL (2020). Threats of bots and other bad actors to data quality following research participant recruitment through social media: cross-sectional questionnaire. J Med Internet Res.

[3] Glazer JV, MacDonnell K, Frederick C, Ingersoll K, Ritterband LM (2021). Liar! Liar! Identifying eligibility fraud by applicants in digital health research. Internet Interv.

[4] Bybee S, Cloyes K, Ellington L, Baucom B, Supiano K, Mooney K (2022). Bots and nots: safeguarding online survey research with underrepresented and diverse populations. Psychol Sex.

[5] Instagram from Meta. Instagram.

[6] Meta | Facebook. Facebook.

[7] Happening now. XCorp.

[8] Newman A, Bavik YL, Mount M, Shao B (2021). Data collection via online platforms: challenges and recommendations for future research. Appl Psychol.

[9] Moss AJ, Rosenzweig C, Jaffe SN, Gautam R, Robinson J, Litman L Bots or inattentive humans? Identifying sources of low-quality data in online platforms. PsyArXiv Preprints. Preprint posted online June 11, 2021.

[10] Dennis SA, Goodson BM, Pearson CA (2020). Online worker fraud and evolving threats to the integrity of MTurk data: a discussion of virtual private servers and the limitations of IP-based screening procedures. Behav Res Account.

[11] Lawlor J, Thomas C, Guhin AT, Kenyon K, Lerner MD, Drahota A (2021). Suspicious and fraudulent online survey participation: introducing the REAL framework. Methodol Innov.

[12] Ballard AM, Cardwell T, Young AM (2019). Fraud detection protocol for web-based research among men who have sex with men: development and descriptive evaluation. JMIR Public Health Surveill.

[13] Bush J, Blackwell CW (2022). Social media as a recruitment strategy with transgender-identified individuals: using an ethical lens to direct methodology. J Transcult Nurs.

[14] Campbell CK, Ndukwe S, Dubé K, Sauceda JA, Saberi P (2022). Overcoming challenges of online research: measures to ensure enrollment of eligible participants. J Acquir Immune Defic Syndr.

[15] Godinho A, Schell C, Cunningham JA (2020). Out damn bot, out: recruiting real people into substance use studies on the internet. Subst Abus.

[16] Heffner JL, Watson NL, Dahne J, Croghan I, Kelly MM, McClure JB, Bars M, Thrul J, Meier E (2021). Recognizing and preventing participant deception in online nicotine and tobacco research studies: suggested tactics and a call to action. Nicotine Tob Res.

[17] Hohn KL, Braswell AA, DeVita JM (2022). Preventing and protecting against internet research fraud in anonymous web-based research: protocol for the development and implementation of an anonymous web-based data integrity plan. JMIR Res Protoc.

[18] Levi R, Ridberg R, Akers M, Seligman H (2022). Survey fraud and the integrity of web-based survey research. Am J Health Promot.

[19] Salinas MR (2023). Are your participants real? Dealing with fraud in recruiting older adults online. West J Nurs Res.

[20] Reagan L, Nowlin SY, Birdsall SB, Gabbay J, Vorderstrasse A, Johnson C, D'Eramo Melkus G (2019). Integrative review of recruitment of research participants through Facebook. Nurs Res.

[21] Ellington M, Connelly J, Clayton P, Collazo-Velazquez C, Lorenzo Y, Trak-Fellermeier MA, Palacios C (2021). A systematic review of the use of social media for recruitment of participants in nutrition, obesity, and physical activity related studies. Curr Dev Nutr.

[22] Burnette CB, Luzier JL, Bennett BL, Weisenmuller CM, Kerr P, Martin S, Keener J, Calderwood L (2022). Concerns and recommendations for using Amazon MTurk for eating disorder research. Int J Eat Disord.

[23] Guest JL, Adam E, Lucas IL, Chandler CJ, Filipowicz R, Luisi N, Gravens L, Leung K, Chavanduka T, Bonar EE, Bauermeister JA, Stephenson R, Sullivan PS (2021). Methods for authenticating participants in fully web-based mobile app trials from the iReach project: cross-sectional study. JMIR Mhealth Uhealth.

[24] Teitcher JE, Bockting WO, Bauermeister JA, Hoefer CJ, Miner MH, Klitzman RL (2015). Detecting, preventing, and responding to "fraudsters" in internet research: ethics and tradeoffs. J Law Med Ethics.

[25] Hardesty JJ, Crespi E, Nian Q, Sinamo JK, Breland AB, Eissenberg T, Welding K, Kennedy RD, Cohen JE (2023). The vaping and patterns of e-cigarette use research study: protocol for a web-based cohort study. JMIR Res Protoc.

[26] Storozuk A, Ashley M, Delage V, Maloney EA (2020). Got bots? Practical recommendations to protect online survey data from bot attacks. Quant Methods Psychol.

[27] Wang J, Calderon G, Hager ER, Edwards LV, Berry AA, Liu Y, Dinh J, Summers AC, Connor KA, Collins ME, Prichett L, Marshall BR, Johnson SB (2023). Identifying and preventing fraudulent responses in online public health surveys: lessons learned during the COVID-19 pandemic. PLOS Glob Public Health.

[28] Vasileiou K, Barnett J, Thorpe S, Young T (2018). Characterising and justifying sample size sufficiency in interview-based studies: systematic analysis of qualitative health research over a 15-year period. BMC Med Res Methodol.

[29] REDCap: Research Electronic Data Capture homepage. REDCap: Research Electronic Data Capture.

[30] Harris PA, Taylor R, Thielke R, Payne J, Gonzalez N, Conde JG (2009). Research electronic data capture (REDCap)--a metadata-driven methodology and workflow process for providing translational research informatics support. J Biomed Inform.

[31] Harris PA, Taylor R, Minor BL, Elliott V, Fernandez M, O'Neal L, McLeod L, Delacqua G, Delacqua F, Kirby J, Duda SN (2019). The REDCap consortium: building an international community of software platform partners. J Biomed Inform.

[32] Graneheim UH, Lundman B (2004). Qualitative content analysis in nursing research: concepts, procedures and measures to achieve trustworthiness. Nurse Educ Today.

[33] Elo S, Kyngäs H (2008). The qualitative content analysis process. J Adv Nurs.

[34] Vaismoradi M, Turunen H, Bondas T (2013). Content analysis and thematic analysis: implications for conducting a qualitative descriptive study. Nurs Health Sci.

[35] Welcome to your professional community. LinkedIn.

[36] Honeybee Hub homepage. Honeybee Hub.

[37] Qualtrics XM: the leading experience management software. Qualtrics XM.

[38] CAPTCHA: telling humans and computers apart automatically. Carnegie Mellon University.

[39] Piehlmaier D (2022). Bot Detection in Online Studies and Experiments.

[40] Goodrich B, Fenton M, Penn J, Bovay J, Mountain T (2023). Battling bots: experiences and strategies to mitigate fraudulent responses in online surveys. Appl Econ Perspect Policy.

[41] What is a web crawler? | How web spiders work. Cloudflare.

[42] Gow G (2021). The scary truth behind the FBI warning: deepfake fraud is here and it’s serious—we are not prepared for an attack. Forbes.

[43] Brainard J, Killett A, Houghton J, Bunn D, Watts L, Mumford S, O'Brien SJ, Lane K The wasps are clever: keeping out and finding bot answers in internet surveys used for health research. Preprints.

[44] Pasternak O (2019). Market research fraud – distributed survey farms exposed. Persona.ly.

[45] Roehl JM, Harland DJ (2022). Imposter participants: overcoming methodological challenges related to balancing participant privacy with data quality when using online recruitment and data collection. Qual Report.

[46] Mournet AM, Kleiman EM (2023). Internet-based mental health survey research: navigating internet bots on Reddit. Cyberpsychol Behav Soc Netw.

[47] Parks AM, Duffecy J, McCabe JE, Blankstein Breman R, Milgrom J, Hirshler Y, Gemmill AW, Segre LS, Felder JN, Uscher-Pines L (2022). Lessons learned recruiting and retaining pregnant and postpartum individuals in digital trials: viewpoint. JMIR Pediatr Parent.

[48] Mitchell JW, Chavanduka TM, Sullivan S, Stephenson R (2020). Recommendations from a descriptive evaluation to improve screening procedures for web-based studies with couples: cross-sectional study. JMIR Public Health Surveill.

[49] Kensington-Miller B (2017). Surviving the first year: new academics flourishing in a multidisciplinary community of practice with peer mentoring. Prof Dev Educ.

[50] Cantin CM, Brune S, Killam L, Glass T, Walker R, Vanderlee E (2022). Establishing a community of practice for doctoral studies amidst the COVID-19 pandemic. Qual Adv Nurs Educ.

[51] McLoughlin C, Patel KD, O'Callaghan T, Reeves S (2018). The use of virtual communities of practice to improve interprofessional collaboration and education: findings from an integrated review. J Interprof Care.

