# Supplementary material for: Evaluating the Problem of Fraudulent Participants in Health Care Research: Multimethod Pilot Study
Source: JMIR Form Res. 2024 Jun 4;8:e51530. doi: 10.2196/51530 (PMC11185910; doi:10.2196/51530)
Supplement: Multimedia Appendix 1 [file formative_v8i1e51530_app1.docx]

| Key areas | Fraud indicators | Supporting quotes |
| --- | --- | --- |
| Identity irregularities | - Unusual names, generic names in line with stereotypical Western names, or names of famous people (eg, John Smith and Britney Spears) - Names not matching the names in their email address - Different names used throughout the email thread - Different names used in email interactions - Different birth dates provided across longitudinal surveys (eg, change in birth date and year) - Multiple signatures on consent forms | - Consent forms - “And I think of the 200, 270 respondents a hundred came back with the signed consent form, but within that as well, we had multiple signatures, the same signatures, different e-mails on that same, so different people were sending me the same consent form but the different email address.” [Research administrator] |
| Unusual participant behaviors | - Attempts to negotiate the type of incentive or the method of incentive delivery - Failure to provide mailing address despite it being required for incentive delivery - Attempts to schedule the interview very quickly, contrary to typical behavior of busy health care professionals - Demonstration of poor knowledge of the study topic - Lack of interest in the interview - Not turning on the camera during interviews | - Negotiations   - “They also asked if I could instead, we promised them a Tim Horton’s and Starbucks gift card and they said that those are not, we don’t want, I don’t want those, we, I get an Amazon gift card instead.” [Research administrator] - Scheduling interviews   - “And my research coordinator noticed that they were responding very, very quickly to schedule the interview, which was quite strange because typically healthcare professionals are really busy and it’s like, it’s a long time to get responses from them. And then when they actually have time to schedule the interview, it’s like a week or a few weeks in advance.” [Associate professor] - Lack of interest   - “But just in speaking with our research coordinator about the interactions that she’s had with people, it kind of like—the way that they’re acting, sort of saying funny things, joking around, laughing in the background. Sometimes she can hear more than one person there and then they’ll just be like, oh, forget it and log off. It really has this kind of Halloween vibe.” [Assistant professor] - Camera off   - “Then when we get to the interview with people, they’ll leave their cameras off during interviews. They will say that they’re child with cancer, but they have obviously an adult voice, but their camera is off.” [Assistant professor] |
| Location discrepancies | - Location of residence not matching participant’s postal code, IP address, or phone number | - Location data   - “And then when you match up the location and the postal code, sometimes there’s a mismatch in terms of they say that they’re in Toronto, but then the post code starts is the V, which is in out of Vancouver.” [Research coordinator]   - “So, people would say, oh, I’m in Ontario, but their IP address was in Texas or their IP address was in Nigeria or something like that. So that was another method that we were able to use.” [Research officer] |
| Suspicious email communication | - Provision of temporary email addresses - Inconsistencies between participant’s name and name used in the email address - Surge of emails in a very short time frame - Clustering of emails around the same time or in close succession - High volume of emails with a similar address structure, such as first name and last name followed by numbers - Receipt of emails at unusual times of the day, such as during the middle of the night - Presence of near-identical wording across emails from different email addresses - Unusual brevity and directness in emails, lacking contextual information such as where the participant saw the advertisement or how they met the study criteria, and minimal salutations or greetings - Lengthy and convoluted emails or emails with gibberish content (eg, Chinese characters with no logical meaning) - Use of different names by the participant throughout the email correspondence | - Unusual brevity   - “...the content of the email that doesn’t really seem to jive, lots of typos, this type of thing. The email being very quick and not, doesn’t really seem to address the point of the study.” [Assistant professor] - Email surges   - “And then we realized there would be a huge influx in emails at certain hours of the day, and it was typically 2:00 to 3:00 AM and all the email addresses were structured the exact same way. So, it would’ve been a generic first name, last name, so Brian James or things like that. And then a series of numbers@gmail.com and everything was the exact same.” [Research officer] - Different names   - “And then we started to realize sometimes they would forget to change the name or in one email they would say, my name’s Kristen. And then in the follow up they would say Nancy, for example. So, there were just these little inconsistencies.” [Research officer] |
| Suspicious responses | - Use of terminology not commonly used by legitimate participants, such as outdated or inappropriate language - General and nonspecific responses that lack the expected level of detail from individuals in the target population - Responses that do not align with the typical patterns of communication or knowledge expected from individuals in the sample population - Consent forms with similar labels or multiple signatures with different names - Responses that are unrelated to the question being asked - Inappropriate responses, including the use of profanity or sexual references - Multiple survey questions left unanswered with no contact information provided - Signs of disinterest or lack of engagement during the study interview | - Uncommon terminology   - “‘Tribe’ is a very outdated term to use for describing Indigenous communities.” [Research assistant] - Unanswered survey questions   - “We have people clicking on the link to the survey and it seems as if they’re stopping at the yes and no questions. So, we have two yes and no questions in the beginning and they would just answer yes and then stop after that. It’s just nothing. It’s just blank. So, there’s no email, there’s no address, there’s no way of reaching out to them.” [Research administrator] |
| Unlikely circumstances | - Disproportionate number of participants self-identifying as belonging to rare or marginalized groups | - Rare or marginalized groups   - “So to have that many people who were identifying as black and Indigenous, both, and also all in this age group of 30 to 39, and also parents...So to get kind of young parents that all had these identifiers, something just kind of did not sit right.” [Assistant professor] |
